# Rectal carcinoma arising in a patient with intestinal and hepatic schistosomiasis due to *Schistosoma mekongi*

**DOI:** 10.1016/j.idcr.2022.e01383

**Published:** 2022-01-06

**Authors:** Matthew Burky, Dimitri Trembath, Christine Bookhout

**Affiliations:** Department of Pathology, Division of Surgical Pathology, Women’s and Children’s Hospitals, The University of North Carolina at Chapel Hill, 3rd Floor, Room 30149, 101 Manning Drive, Chapel Hill 27514, NC, USA

**Keywords:** Schistosomiasis, *Schistosoma mekongi*, Rectal carcinoma, Hepatic fibrosis, Non-cirrhotic portal hypertension, HNPCC, hereditary non-polyposis colorectal cancer, ELISA, enzyme-linked immunosorbent assay, CCA, circulating cathodic antigen, CAA, circulating anodic antigen, PCR, polymerase chain reaction

## Abstract

Schistosomiasis is a parasitic trematode infection spread by snails with multiple species causing human disease. Infection can cause liver disease, including fibrosis and portal hypertension, and has been linked to malignancies such as bladder and colorectal cancer. We describe a case of *Schistosoma mekongi,* a geographically limited form of schistosomiasis, in a Laotian immigrant who presented with both hepatic fibrosis and rectal cancer, with numerous schistosome eggs present in the patient’s rectal resection. We believe this case is the first report of a rectal carcinoma arising in the setting of *S. mekongi* infection.

## Introduction

**Fig. 1:**
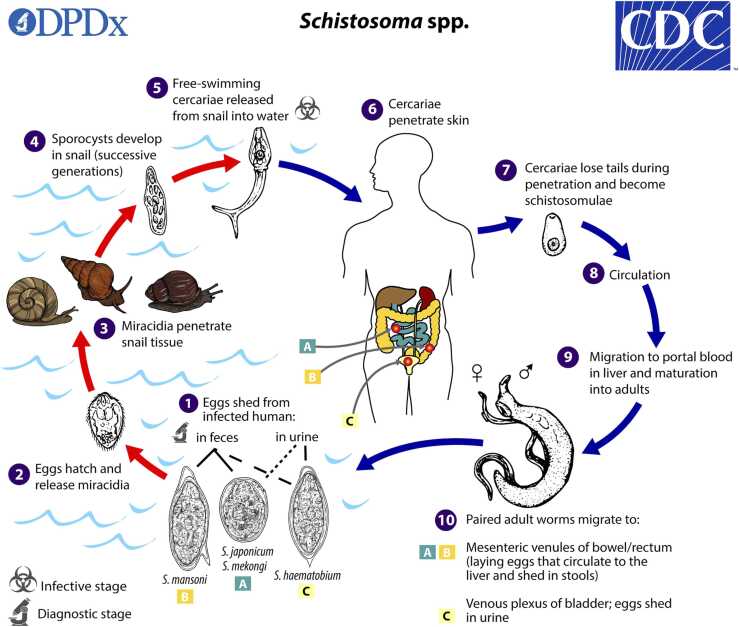
Life cycle of *Schistosoma* species. da Silva, PhD; Melanie Moser.

Reproduced with permission from CDC.gov Public Health Image Library (CDC / Alexander J) [24].

Schistosomiasis, also known as bilharzia, is a parasitic disease caused by blood trematodes (flukes) in the genus *Schistosoma* and spread by snail intermediate hosts (Fig. 1). Three species are responsible for the majority of disease in humans, *Schistosoma haematobium, S. japonicum*, and *S. mansoni*, and by conservative estimates at least 230 million people worldwide are believed to be infected [1], [2]. Additional species exist with a more limited geographic range, including *Schistosoma mekongi*, which is localized to the Mekong River and its tributaries in Laos, Cambodia, and Thailand [2], [3], [4]. *S. mekongi* is spread by the snail intermediate host *Neotricula aperta*, and definitive mammalian hosts include dogs and pigs as well as humans [2], [5].

Schistosomes live in the mesenteric veins of human hosts for years, and produce large numbers of eggs that are either excreted or retained in host tissues where they can induce inflammatory and granulomatous responses [1], [2]. *S. mekongi* can be diagnosed by rectal biopsy, since the rectum is an accessible site where eggs can be identified in tissue, as well as by identification of eggs in stool [6], [7].

Schistosomiasis is a common cause of liver disease in endemic areas, and *S. mekongi* is associated with periportal fibrosis and non-cirrhotic portal hypertension. The liver disease is due to inflammation and fibrosis in the portal areas of the liver, believed to be a reaction to eggs lodged within host tissue causing chronic granulomatous inflammation [8], [9], [10], [11]. Although treatment can kill adult worms and prevent additional egg deposition, it cannot remove eggs that are already within tissue. Nor can treatment entirely reverse fibrosis which has already occurred, although some degree of regression of fibrosis and portal hypertension may occur over time [10].

Schistosomiasis is also associated with malignancy, with perhaps the best established connection between *S. haematobium* and squamous cell carcinoma of the bladder [12], [13]. A relationship between schistosomiasis and colorectal carcinoma has also been suggested in the literature, but is less universally accepted; evidence appears strongest for a link between *S. japonicum* and colorectal cancer in Southeast Asia [13], [14], [15], [16]. Intestinal involvement is common in schistosomiasis infection, with egg deposition and associated granulomatous inflammation leading to chronic colitis, and has been associated with polyp formation [10], [16]. It is hypothesized that this ongoing chronic inflammation may predispose patients to develop colorectal carcinoma, in a similar fashion to the increase in risk in inflammatory bowel disease. However, although *S. mekongi* is known to involve the colorectum, we did not identify studies assessing its association with colorectal cancer. In this paper, we present a case of *Schistosoma mekongi* associated with invasive rectal adenocarcinoma.

## Case report

The patient is a Laotian woman in her late 40 s who had been living in the US for approximately 20 years. She grew up in Laos and spent significant time in the Mekong River. She reported episodes of jaundice as a child as well as abnormal liver function tests during pregnancy. Subsequently, she was diagnosed with schistosomiasis and treated; however, she was found to have chronic liver disease with fibrosis, portal hypertension, splenomegaly, and esophageal varices. Although she was listed for liver transplantation, her MELD score did not progress to a level of high priority. During this period, she had several episodes of bacteremia and sepsis which were treated with antibiotics, but otherwise her liver disease remained relatively stable.

She began to experience rectal bleeding and proctoscopy revealed a low rectal mass, with biopsy showing adenocarcinoma. Imaging suggested that the mass was locally advanced with possible invasion through the rectum into adjacent structures. Calcification along the portal venous system was also noted on imaging. She underwent neoadjuvant chemoradiation followed by surgery (total mesorectal excision). Pathology showed invasive colorectal adenocarcinoma with invasion into but not through the muscularis propria and one lymph node positive for metastatic carcinoma, stage ypT2 pN1a. The tumor showed intact expression of mismatch repair enzymes, which did not provide evidence for a diagnosis of hereditary non-polyposis colorectal cancer (HNPCC).

**Fig. 2:**
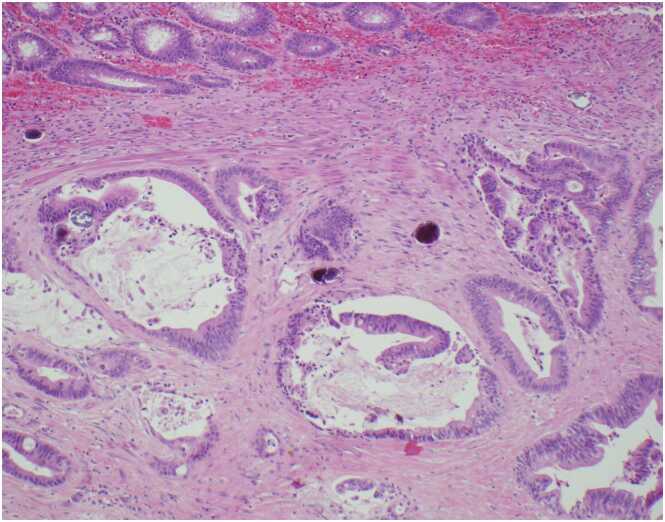
100x image of rectal adenocarcinoma with submucosal invasion and calcified *Schistosoma mekongi* eggs.

**Fig. 3:**
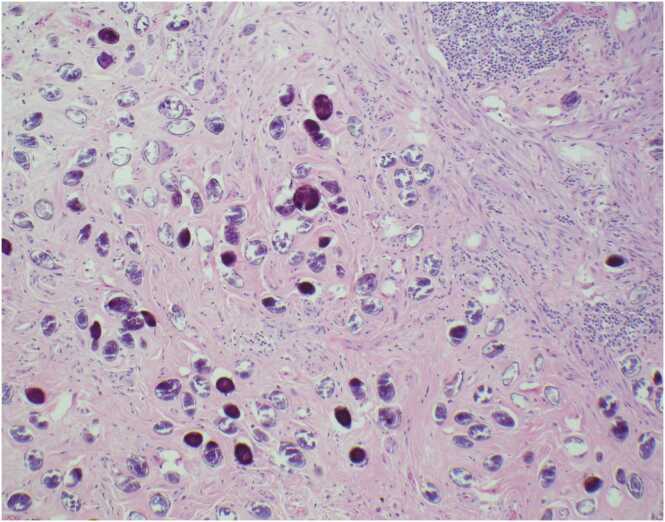
100x image of abundant calcified *Schistosoma mekongi* eggs with associated chronic inflammation.

**Fig. 4:**
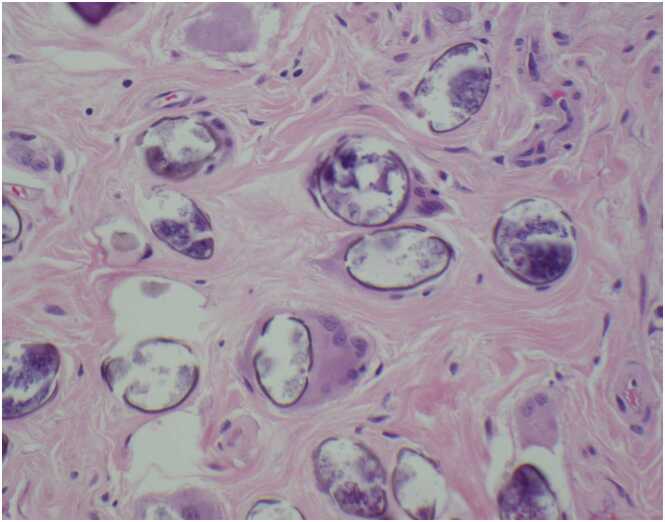
400x image of *Schistosoma mekongi* eggs with associated foreign body giant cell response.

The colonic resection was also notable for numerous ovoid calcified fragments consistent with schistosome eggs, predominated located in the colonic submucosa. The eggs lacked a pronounced spine and measured approximately 40–50 by 50–60 µm in size. Given the patient’s history of exposure to the Mekong River and the egg morphology, the findings were considered diagnostic of *S. mekongi*. A large number of eggs were present, and there were areas of associated chronic and histiocytic inflammation and foreign body giant cell reaction (Fig. 2, Fig. 3, Fig. 4). Schistosome eggs were also identified within pericolonic lymph node tissue.

After an initial period of remission, the patient developed local recurrence within 2 years, followed by metastatic disease including multiple pulmonary nodules. Unfortunately, despite chemotherapeutic treatment, she passed away in her early 50 s

## Discussion

Our patient presented with a relatively classic history for *Schistosoma mekongi* infection, including exposure to the waters of the Mekong River in childhood. Her original diagnosis was made at an outside facility, and information was not available regarding how the diagnosis was made. Active schistosomiasis is usually diagnosed by identification of eggs in urine (*S. haematobium*) or stool (*S. japonicum, S. mansoni*, and *S. mekongi*), or by tissue biopsies, with the rectum considered an accessible site for biopsy [2]. However, the sensitivity of these tests is not optimal. Given the quantity of eggs in the submucosa of our patient’s rectal resection, rectal biopsy would have had a good chance of yielding a diagnosis. Newer methods being developed for diagnosis of *S. mekongi* include antibody detection using an enzyme-linked immunosorbent assay (ELISA) and schistosome circulating antigen detection (cathodic and anodic circulating antigens (CCA, CAA)) in urine and serum, which have showed higher rates of positivity than stool tests in Laos and Cambodia [17]. Antigens directed against *Schistosoma mansoni* have also been shown to cross react with and detect *S. mekongi*-directed antibodies [18]. While serologic tests can be useful in travelers, they are of limited use in endemic areas because they cannot determine between past and active infection, although circulating antigen detection remains useful [2]. Molecular methods such as polymerase chain reaction (PCR) testing also show promise for diagnosis of schistosomiasis.

*S. mekongi*, as well as other forms of schistosomiasis, is generally treated with the drug praziquantel [19], [20]. Single dose cure rates for *S. mekongi* in one study of schoolchildren were 75.0% and 80.8% for 40 mg/kg and 75 mg/kg doses [19]. Given the prevalence of this condition in the Mekong River basin and association with severe liver disease, mass treatment was been a public health aim. In one study, seven rounds of mass treatment with praziquantel were administered between 1995 and 2002 in Cambodia, which reportedly reduced infection rate from 50% to below 3%, although liver disease at the end of the treatment period was still markedly more frequent in endemic areas than in controls in non-endemic areas [8]. Similar mass treatment was initiated in Laos in the late 1980 s, although program interruptions have been an issue [4]. Eventual eradication of *S. mekongi* has been proposed as a goal, although the existence of other mammalian hosts including pigs and dogs complicates eradication efforts. Our patient was likely infected in childhood, and reported a history of liver disease in other relatives in Laos also presumably related to schistosomiasis. She was treated prior to arrival at our institution, and likely did not have active disease at the time of presentation.

Our patient showed classic clinical and radiologic findings of schistosomiasis-associated liver disease, although liver biopsy was not performed at our institution. She had evidence of fibrosis and portal hypertension, as well as esophageal varices and splenomegaly. Liver damage in schistosomiasis is believed to be a reaction to the parasite eggs causing inflammation and fibrosis in the presinusoidal portal areas and impeding blood flow through the portal venous system. The resulting portal hypertension leads to splenomegaly and evidence of porto-systemic collateral circulation such as esophageal varices [9]. Interestingly, although the liver may appear nodular due to fibrosis, hepatocellular function usually remains intact since the hepatic arterial blood supply is not impeded [9]. Therefore, the liver disease is a form of non-cirrhotic portal hypertension rather than true cirrhosis, although some of our patient’s imaging notes inaccurately characterized her liver as cirrhotic. Given that normal liver architecture is generally preserved with retention of lobular architecture, the fibrosis may be somewhat more reversible after anti-parasitic treatment than that of classic cirrhosis [10]. *S. mansoni, S. japonicum*, and *S. mekongi* are believed to have a stronger association with liver disease than *S. hematobium* [9], and may also be associated with hepatocellular carcinoma [13]. In a study in Cambodia, evidence of periportal fibrosis with portal hypertension was diagnosed in 46% of adults in an area endemic for *S. mekongi* (versus 0% in non-endemic control areas), and only 11% had normal liver ultrasounds (versus 99% in non-endemic areas) [8]. These findings were noted even after mass treatment, with 93% of people in the endemic area reporting having at least one and 63% having at least three treatments of praziquantel in previous years [8]. Therefore, liver disease due to *S. mekongi* is a serious and frequent issue in endemic areas and in those who have relocated from such areas.

Intestinal involvement is also a known complication of schistosomal infections, including *S. mekongi*, with eggs most commonly lodging in the submucosa of the colon and rectum. Granulomatous inflammation can lead to mucosal ulceration, hyperplastic changes, and polyp formation with schistosome eggs present in the polyps [10]. In the previously described Cambodian study, blood in stool (41 vs. 20%) and abdominal pain (78 vs. 57%) were reported at a higher rate in *S. mekongi* endemic areas than non-endemic control areas. Another study compared 289 schistosomiasis-associated colorectal cancers with 165 cases without schistosomiasis, and found that polyps, pseudopolyps, ectopically proliferating glands, disintegrated muscularis mucosae, denudation, and multicentric carcinoma were more frequently encountered in the schistosomiasis-associated group [16]. The authors proposed that chronic schistosomiasis may predispose patients to colorectal carcinoma in a similar manner to inflammatory bowel disease [16]. The connection between schistosomiasis and colon cancer has often been suggested in the literature, but is not universally accepted [13], [14], [15], [16], [21], [22]. For example, a case-control study in rural China demonstrated an odds ratio of 3.3 for development of colon cancer in patients with chronic *S. japonicum* compared to matched controls with no previous exposure to schistosomal infection [22]. Cancers in schistosomiasis patients may present at a younger age and show a predilection for the rectum [14], [21], both of which would fit with our patient’s clinical presentation. We did not identify studies of colorectal cancer risk specifically related to *S. mekongi* in the literature, and believe that our case is the first report of a rectal carcinoma arising in the setting of *S. mekongi* infection. The only prior case report we identified of a malignancy in the setting of *S. mekongi* was a case of small bowel leiomyosarcoma, although the authors suggest the possibility that the infection may have been coincidental rather than pathogenically associated [23].

## Conclusion

In this report, we describe a case of rectal carcinoma diagnosed in a patient with history of *Schistosoma mekongi* infection and associated liver disease, with numerous *S. mekongi* eggs identified in her rectal resection. We believe that this is the first reported case of a rectal carcinoma arising in the setting of *S. mekongi* infection. While the link between schistosomiasis and cancer is considered well-established in some malignancies (such as *S. haematobium* and bladder cancer), the connection between schistosomiasis and colorectal cancer is not uniformly accepted, although existing evidence seems strongest for *S. japonicum*. Our case raises the possibility of an association between *S. mekongi* and colorectal cancer, and suggests that further study may be warranted and that colorectal cancer screening is important in individuals with a history of *S. mekongi* infection.

## Funding

This research did not receive any specific grant from funding agencies in the public, commercial, or not-for-profit sectors.

## Ethical approval

Not required.

## Consent

Patient is deceased and neither patient identifying information nor images other than non-identifiable microscopic images are included in the report

## References

[1] Gryseels B., Polman K., Clerinx J., Kestens L. (2006). Human schistosomiasis. Lancet.

[2] Colley D.G., Bustinduy A.L., Secor W.E., King C.H. (2014). Human schistosomiasis. Lancet.

[3] Ziegler K., Möller F.W., Detvongea S. (1988). Peculiarities of Mekong schistosomiasis with particular attention to the People’s Democratic Republic of Laos. Bull Inst Marit Trop Med Gdyn.

[4] Muth S., Sayasone S., Odermatt-Biays S., Phompida S., Duong S., Odermatt P. (2010). *Schistosoma mekongi* in Cambodia and Lao People's Democratic Republic. Adv Parasitol.

[5] Strandgaard H., Johansen M.V., Pholsena K., Teixayavong K., Christensen N.O. (2001). The pig as a host for *Schistosoma mekongi* in Laos. J Parasitol.

[6] Lorette G., Jaafar M.R., Grojean M.F., Duong T. (1983). *Schistosomiasis mekongi* diagnosed by rectal biopsy. Br Med J Clin Res Ed.

[7] Wittes R., MacLean J.D., Law C., Lough J.O. (1984). Three cases of *schistosomiasis mekongi* from northern Laos. Am J Trop Med Hyg.

[8] Keang H., Odermatt P., Odermatt-Biays S., Cheam S., Degrémont A., Hatz C. (2007). Liver morbidity due to *Schistosoma mekongi* in Cambodia after seven rounds of mass drug administration. Trans R Soc Trop Med Hyg.

[9] Shaker Y., Samy N., Ashour E. (2014). Hepatobiliary Schistosomiasis. J Clin Transl Hepatol.

[10] Elbaz T., Esmat G. (2013). Hepatic and intestinal schistosomiasis: review. J Adv Res.

[11] Burke M.L., Jones M.K., Gobert G.N., Li Y.S., Ellis M.K., McManus D.P. (2009). Immunopathogenesis of human schistosomiasis. Parasite Immunol.

[12] Schwartz D.A. (1981). Helminths in the induction of cancer II. *Schistosoma haematobium* and bladder cancer. Trop Geogr Med.

[13] Yosry A. (2006). Schistosomiasis and neoplasia. Contrib Microbiol.

[14] H Salim O.E., Hamid H.K., Mekki S.O., Suleiman S.H., Ibrahim S.Z. (2010). Colorectal carcinoma associated with schistosomiasis: a possible causal relationship. World J Surg Oncol.

[15] Feng H., Lu A.G., Zhao X.W., Han D.P., Zhao J.K., Shi L., Schiergens T.S., Lee S.M., Zhang W.P., Thasler W.E. (2015). Comparison of non-schistosomal rectosigmoid cancer and schistosomal rectosigmoid cancer. World J Gastroenterol.

[16] Ming-Chai C., Chi-Yuan C., Pei-Yu C., Jen-Chun H. (1980). Evolution of colorectal cancer in schistosomiasis: transitional mucosal changes adjacent to large intestinal carcinoma in colectomy specimens. Cancer.

[17] Vonghachack Y., Sayasone S., Khieu V., Bergquist R., van Dam G.J., Hoekstra P.T., Corstjens P., Nickel B., Marti H., Utzinger J., Muth S., Odermatt P. (2017). Comparison of novel and standard diagnostic tools for the detection of *Schistosoma mekongi* infection in Lao People's Democratic Republic and Cambodia. Infect Dis Poverty.

[18] Nickel B., Sayasone S., Vonghachack Y., Odermatt P., Marti H. (2015). *Schistosoma mansoni* antigen detects *Schistosoma mekongi* infection. Acta Trop.

[19] Lovis L., Mak T.K., Phongluxa K., Ayé Soukhathammavong P., Vonghachack Y., Keiser J., Vounatsou P., Tanner M., Hatz C., Utzinger J., Odermatt P., Akkhavong K. (2012). Efficacy of praziquantel against *Schistosoma mekongi* and *Opisthorchis viverrini*: a randomized, single-blinded dose-comparison trial. PLoS Negl Trop Dis.

[20] Nash T.E., Hofstetter M., Cheever A.W., Ottesen E.A. (1982). Treatment of *Schistosoma mekongi* with praziquantel: a double-blind study. Am J Trop Med Hyg.

[21] Matsuda K., Masaki T., Ishii S., Yamashita H., Watanabe T., Nagawa H., Muto T., Hirata Y., Kimura K., Kojima S. (1999). Possible associations of rectal carcinoma with *Schistosoma japonicum* infection and membranous nephropathy: a case report with a review. Jpn J Clin Oncol.

[22] Qiu D.C., Hubbard A.E., Zhong B., Zhang Y., Spear R.C. (2005). A matched, case-control study of the association between *Schistosoma japonicum* and liver and colon cancers, in rural China. Ann Trop Med Parasitol.

[23] Cuesta R.A., Kaw Y.T., Duwaji M.S. (1992). *Schistosoma mekongi* infection in a leiomyosarcoma of the small bowel: a case report. Hum Pathol.

[24] Centers for Disease Control and Prevention (CDC) (2019). https://www.cdc.gov/dpdx/schistosomiasis/index.html.

